# Copper Efflux System Required in Murine Lung Infection by Haemophilus influenzae Composed of a Canonical ATPase Gene and Tandem Chaperone Gene Copies

**DOI:** 10.1128/iai.00091-23

**Published:** 2023-04-04

**Authors:** Sandy M. Wong, Jeffrey Gawronski, Brian J. Akerley

**Affiliations:** a Department of Microbiology and Immunology, University of Massachusetts Medical School, Worcester, Massachusetts, USA; b Department of Microbiology and Immunology, University of Mississippi Medical Center, Jackson, Mississippi, USA; c Department of Cell and Molecular Biology, Center for Immunology and Microbial Research, University of Mississippi Medical Center, Jackson, Mississippi, USA; University of California, Davis

**Keywords:** *Haemophilus influenzae*, NTHi, copper, *copA*, *copZ*, lung infection, mouse model, antimicrobial agents, efflux pumps

## Abstract

Copper is an essential micronutrient but is toxic at high concentrations. In Haemophilus influenzae mechanisms of copper resistance and its role in pathogenesis are unknown; however, our previous genetic screen by transposon insertion-site sequencing implicated a putative cation transporting ATPase (*copA*) in survival in a mouse lung infection model. Here, we demonstrate that H. influenzae *copA* (HI0290) is responsible for copper homeostasis involving the *merR*-type regulator, *cueR*, as well as six tandem copies of the metallochaperone gene, *copZ*. Deletion of the ATPase and metallochaperone genes resulted in increased sensitivity to copper but not to cobalt, zinc, or manganese. Nontypeable H. influenzae (NTHi) clinical isolate NT127 has the same locus organization but with three copies of *copZ*. We showed that expression of the NTHi *copZA* operon is activated by copper under the regulatory control of CueR. NTHi single *copA* and *copZ* mutants and, especially, the double deletion *copZA* mutant exhibited decreased copper tolerance, and the Δ*copZA* mutant accumulated 97% more copper than the wild type when grown in the presence of 0.5 mM copper sulfate. Mutants of NT127 deleted of the ATPase (*copA*) alone and deleted of both the ATPase and chaperones (*copZ1-3*) were 4-fold and 20-fold underrepresented compared to the parent strain during mixed-infection lung challenge, respectively. Complementation of *cop* locus deletion mutations restored copper resistance and virulence properties. NTHi likely encounters copper as a host defense mechanism during lung infection, and our results indicate that the *cop* system encodes an important countermeasure to alleviate copper toxicity.

## INTRODUCTION

Nontypeable Haemophilus influenzae (NTHi) is a gram-negative bacterial pathogen that colonizes the nasopharynx of healthy humans. As a commensal microbe, NTHi is commonly isolated from the upper airways of the human host with a carriage frequency of approximately 20 to 80%. However, dissemination to other anatomical locations results in a range of infections, including otitis media, sinusitis, pneumonia, and exacerbations of chronic obstructive pulmonary disease (COPD). In COPD, NTHi is a leading cause of acute lung infections that accelerate disease progression, causing ~50% of bacteria-mediated exacerbations and ~30% of all COPD exacerbations (1–5).

In our previous studies we utilized genome-wide mutant fitness analyses to identify factors of H. influenzae required for growth and survival in the mouse lung model (6, 7). Our approach, termed HITS (high-throughput insertion tracking by deep sequencing), in which transposon-chromosome junctions are identified and enumerated from transposon mutant libraries by high-throughput DNA sequencing, revealed a core set of genes required for survival in this site. These include known virulence determinants as well as genes in diverse processes, including metal transport (e.g., copper, zinc), for nutrient acquisition and efflux that were previously unrecognized to play a role in pathogenesis in this organism. In fact, validation of two genes with putative transport functions from our HITS screen led to the discovery of a novel zinc transport system crucial for NTHi survival in the mouse lung (8).

Copper is an essential micronutrient and serves as a biological cofactor yet is toxic in excess. All living organisms have developed strategies to handle the toxic effects of copper in the cell. Bacteria possess dedicated systems to maintain copper homeostasis and ensure the appropriate balance of intracellular levels (9–11). Copper is a necessary cofactor for certain respiratory complexes (redox enzymes) (9), but toxic doses can also cause degradation of Fe-S clusters in vital enzymes of key biosynthetic pathways leading to impaired cell function (12).

To prevent accumulation of toxic copper concentrations, excess amounts must be detoxified and exported out of the cell, and diverse canonical copper efflux mechanisms implicated in this function have been identified among bacteria. Copper homeostasis involves at least three main proteins, a copper-exporting ATPase (Cu ATPase) to remove copper from the cytosol, a copper chaperone that delivers copper to the Cu ATPase, and a metalloregulator that modulates activity of the Cu ATPase and chaperone (9–11). Many gram-negative bacteria (e.g., Escherichia coli, Pseudomonas spp.) possess additional systems for removing copper from the periplasm and out into the extracellular space (9, 10, 13). Studies of mechanisms of copper transport and homeostasis in these two prototypical model organisms indicate that they have multiple copper efflux systems with seemingly redundant functions. Two are chromosomally encoded: the primary copper efflux system, *cue*, which consists of a copper exporter (CopA), periplasmic oxidase (CueO), and copper-responsive metalloregulator (CueR), and the *cus* system (CusCFBA or CusCBA), which is involved in periplasmic copper efflux. The third is a plasmid-encoded system containing a six- to seven-gene operon (Pco or Cop system) also involved in periplasmic copper handling (10, 14–16). Most *Proteobacteria* have homologs or variations of the Cue, Cus, or Pco (Cop) systems.

Emerging evidence indicates that the innate immune response utilizes the toxic properties of copper to defend against infectious microorganisms (17, 18). While the mechanism by which copper exerts antimicrobial activity is not well understood, copper tolerance has been recognized as an important virulence mechanism in pathogenic bacteria (19). The role of *copA* alone or in combination with other copper resistance genes has been evaluated in animal models of infection for numerous pathogenic gram-negative and gram-positive bacteria, and *copA* was found to be required for virulence in some bacteria (Acinetobacter baumannii, Klebsiella pneumoniae, Streptococcus pneumoniae, Pseudomonas aeruginosa, Streptococcus agalactiae and Salmonella enterica serovar Typhimurium) (20–26), but not in all cases. In some studies deletion of *copA* alone (Helicobacter pylori, Listeria monocytogenes, Streptococcus suis, Streptococcus pyogenes, Actinobacillus pleuropneumoniae) (27–31) or deletion in combination with a gene encoding copper amine oxidase in Vibrio cholerae (32) was inconsequential for *in vivo* virulence. Moreover, in some species, *copA* was differentially required for colonization in distinct tissue sites in mice (P. aeruginosa, S. agalactiae) (24, 25). Regardless of whether *copA* was required for virulence *in vivo*, *copA* was needed for copper tolerance *in vitro* when tested in the studies cited above, and these studies suggest that the role of specific copper tolerance factors during pathogenesis may differ depending on the constellation of systems present in each species.

In the genome of H. influenzae Rd, HI0290, HI0291-2, and HI0293 are annotated as a putative copper-transporting ATPase, copper chaperones, and a copper efflux regulator, respectively, but no gene encoding the multicopper oxidase CueO is evident, nor are genes encoding the Cus or plasmid-encoded Pco systems. H. influenzae appears to have a simplified copper efflux system which could provide an opportunity to examine this potential virulence feature in a more streamlined context compared to that of organisms with multiple copper resistance pathways. The objective of this study was to characterize the system responsible for copper homeostasis in H. influenzae, for which no studies on copper tolerance have yet been reported, and apply this information to further examine its role in NTHi pathogenesis. The results of this study suggest that copper tolerance is an important virulence mechanism for NTHi in the lung and that the copper efflux system is required for optimal survival in this niche.

## RESULTS

### The genomic organization of the *cop* locus in H. influenzae shows variable copies of the metallochaperone gene.

In our previous genetic screen using the HITS method (6, 7), we identified a putative H. influenzae *copA* (locus HI0290 when mapped to reference strain KW20) as likely to be essential for growth and survival in a mouse lung infection model. In this screen, abundant transposon insertions in HI0290 (called *copA* here) present in our input H. influenzae mutant library were underrepresented in the population recovered after infection with this mutant library in the mouse lung model (see Fig. S1 in the supplemental material) (6). Bioinformatic analysis suggests that *copA* encodes a copper-transporting P_1B_-type ATPase that effluxes Cu^+^ across the inner membrane and plays a role in copper homeostasis and tolerance (33, 34). The predicted CopA_Hi_ is a 722-amino acid protein with a single N-terminal metal binding domain (N-MBD) and 8 predicted transmembrane-spanning helices. The signature sequence motifs of CPCX_6_P in helix 6 (where X is any amino acid), YN in helix 7, and MX_2_SS in helix 8 are strictly conserved in P_1B-1_-ATPases, suggesting the presence of these residues as key determinants for copper specificity (34). For example, H. influenzae CopA has 38% identity/57% similarity to CopA1 of Pseudomonas aeruginosa (PA3920) and 37% identity/55% similarity to E. coli CopA (b0484). In H. influenzae genomes, examination of the DNA regions surrounding *copA* revealed genes that are likely involved in copper tolerance. Several metallochaperone genes (called *copZ* here) are located directly upstream of *copA* in an apparent operon, and this operon is likely controlled by the proximal MerR-like transcriptional regulator (called CueR here) (Fig. S1). The putative H. influenzae CopZ metallochaperones have 41% identity and 65% similarity to the well-characterized Cu(I)-binding protein CopZ of Bacillus subtilis (BSU33510) and display the conserved metal-binding CXXC motif (35–38). CopZ chaperones function to traffic copper ions to the ATPase and buffer against the toxic effects of copper in the cytoplasm (38–40). The amino acid sequence of the H. influenzae regulator CueR is highly similar to that of the copper-responsive CueR which controls *copA* expression in many gram-negative bacteria (41, 42). For example, CueR_Hi_ has 49% identity/67% similarity to CueR of P. aeruginosa (PA4778) and 40% identity/64% similarity to CueR of E. coli (b0487). The CueR_Hi_ sequence possesses the signature motifs of CXGX_5_C for copper specificity in the metal binding loop and SX_2_V motif in the N-terminal region of the dimerization helix (43).

In the draft genome sequences of H. influenzae RdAW (at least 99.98% identical in nucleotide sequence to the reference strain Rd KW20 [6]) and the clinical NTHi isolate NT127, the *cop* locus is represented in several contigs. To determine the genetic organization of the locus in these strains, regions were amplified by PCR using primers that bound to sites in *cueR* and *copA* (Fig. S2). Sequencing of the NT127 and RdAW fragments revealed three copies of the chaperone, designated *copZ1*, *copZ2*, and *copZ3*, and six copies of the chaperone (*copZ1* through *copZ6*), respectively. To examine whether tandem repeats of *copZ* also exist in other H. influenzae strains, we surveyed the length of the operon between *cueR* and *copA* by PCR (Fig. S3). We found variation of *copZ* copy number ranging from three to seven among NTHi isolates, with six copies of *copZ* in Rd KW20 and RdAW rather than the two annotated copies for KW20 in the NCBI database (GenBank accession no. L42023.1).

**FIG 1:**
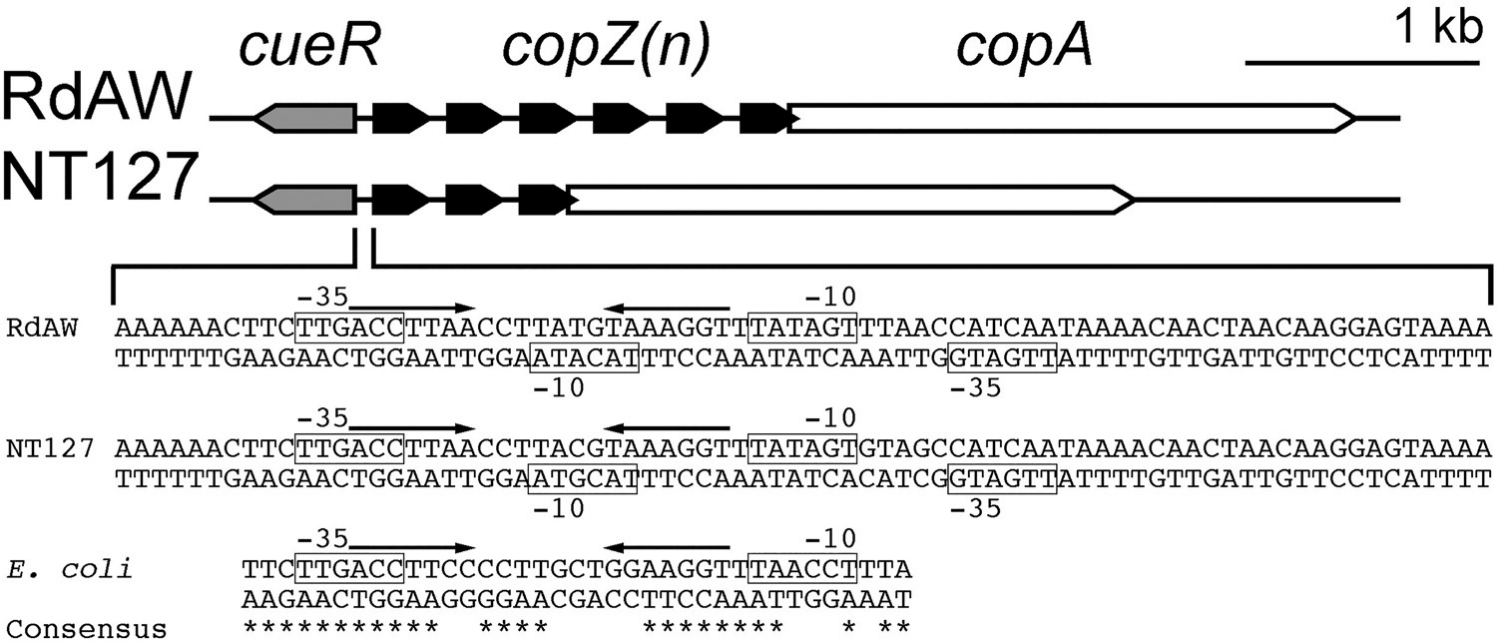
Genomic organization of the *cop* locus in H. influenzae. The locus consists of the regulatory gene *cueR* (gray), multiple copies of the copper chaperone gene *copZ* (black), designated as *copZ*(n) representing *copZ1*, *copZ2*, etc., and the Cu^+^-ATPase gene *copA* (white). The complete intergenic sequence between *cueR* and *copZ1* is shown in H. influenzae. The CueR binding site is an imperfect palindrome interrupted by seven base pairs, ACCTTaA-N7-TaAAGGT (represented by arrows); boxes indicate the putative –10 and –35 elements for P*cop* (top strand) and P*cueR* (bottom strand). Similarly, the features for the E. coli P*cop* are noted (41, 54). The consensus shows identities between H. influenzae sequences and the E. coli *cop* promoter.

Comparison of the amino acid sequences of CopZ copies in both strains reveals three amino acid differences among the two strains that are either conservative or semiconservative substitutions as well as the conserved metal-binding CXXC motif (CGCC) at residues 13 to 16 (Fig. S4). CopZ1 and 3 are identical in amino acid sequence within NT127; CopZ3 through Z6 are identical in amino acid sequence within RdAW (the predicted CopZ proteins HI0292 and HI0291 of Rd KW20 are identical in amino acid sequence to CopZ1 and CopZ3 to Z6 of RdAW, respectively). The genetic organization of the putative *cop* locus in RdAW and NT127 is shown in Fig. 1. The occurrence of multiple nearly identical tandem copies of the copZ chaperone gene appears to be unique to H. influenzae and its close relative Haemophilus haemolyticus (see Discussion).

### Genes of the *cop* locus are required for copper tolerance in H. influenzae.

**FIG 2:**
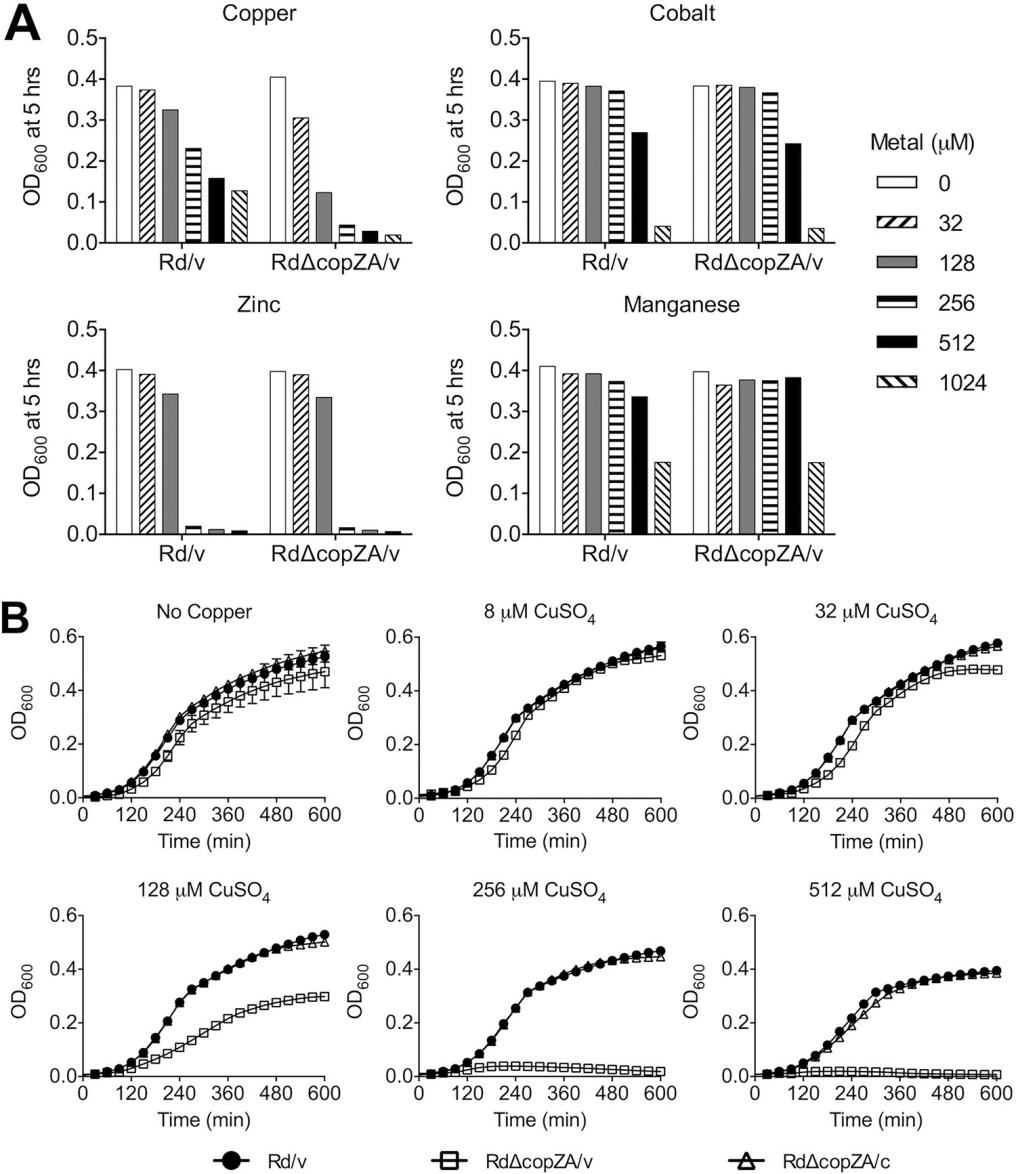
Effect of metal ions on the growth of H. influenzae *cop* mutants. (A) Growth densities of parental Rd/v and mutant RdΔcopZA/v cultures at 5 h of incubation in the presence of various concentrations of Cu^2+^, Co^2+^, Zn^2+^, or Mn^2+^. (B) Growth curve profiles of Rd/v, RdΔcopZA/v, and complemented RdΔcopZA/c strains in copper-supplemented media. Cultures were grown aerobically in sBHI containing the indicated metal ion concentrations in 96-well plates. Panel B represents the averages and standard deviation (SD) of duplicate samples. The errors bars are smaller than the line symbols in most graphs.

The *copA* gene has been shown to be needed for copper tolerance *in vitro* in the model organism E. coli (44) as well as in many bacterial systems mentioned earlier, but this appears not to be the case for Bordetella pertussis (45). In some studies that have evaluated the role of *copZ* in copper resistance, for example in Bacillus subtilis and Streptococcus mutans, *copZ* was shown to contribute to copper tolerance *in vitro* (46, 47), while in Listeria monocytogenes growth of a *copZ* mutant was not sensitive to copper (28). To initially evaluate the importance of *copZA* in copper homeostasis in H. influenzae, first a mutant that contains a precise deletion of the copper chaperones and the ATPase, RdΔcopZA/v, was constructed by gene replacement in RdAW (Table 1) (Materials and Methods). Growth inhibition by metal cations commonly transported by P_1B_ ATPases was assayed in the parental strain Rd/v (48) and mutant RdΔcopZA/v strains (Fig. 2A). Culture densities after 5 h of growth in various concentrations of copper, cobalt, zinc, or manganese showed that Cu^2+^ at concentrations of 32 μM or greater inhibited the growth of the *copZA* mutant relative to the parental strain, Rd/v. Culture densities between both strains were equivalent in the presence of Co^2+^, Zn^2+^, or Mn^2+^. Additionally, the growth kinetic profiles in Fig. 2B showed very little to no growth of the *copZA* mutant in 256 μM and 512 μM CuSO_4_, while complementation of the *copZA* mutant restored growth to parental levels. Therefore, these results implicate the *copZA* operon in copper homeostasis and tolerance in H. influenzae.

**TABLE 1:** H. influenzae strains and plasmids used in this study

Strain or plasmid	Genotype and/or relevant features	Reference or source
Strains		
Rd KW20 ATCC 51907	Nonencapsulated H. influenzae type d	ATCC
RdAW	Nonencapsulated H. influenzae type d	6
Rd/v	RdAW *xylA^Δ4-804^*^::^*^tetAR^*; empty vector pXT10 cloning vector at the *xyl* locus; Tet^r^	48
RdΔcopZA	RdAW Δ*copZA*::*aacC1*; Gm^r^	This study
RdΔcopZA/v	RdAW Δ*copZA* with empty vector pXT10 at the *xyl* locus; Gm^r^ Tet^r^	This study
RdΔcopZA/c	RdAW Δ*copZA* with *copZA* expressed under the endogenous promoter for complementation at the *xyl* locus; Gm^r^ Tet^r^	This study
PittII	Nontypeable H. influenzae clinical isolate	Provided by Garth Ehrlich
PittEE	Nontypeable H. influenzae clinical isolate	Provided by Garth Ehrlich
PittGG	Nontypeable H. influenzae clinical isolate	Provided by Garth Ehrlich
22.4.21	Nontypeable H. influenzae clinical isolate	Provided by Garth Ehrlich
22.1.21	Nontypeable H. influenzae clinical isolate	Provided by Garth Ehrlich
6P18HI	Nontypeable H. influenzae clinical isolate	Provided by Garth Ehrlich
86-028NP	Nontypeable H. influenzae clinical isolate	Provided by Bob Munson
NT127	Nontypeable H. influenzae clinical isolate	6
NTX	NT127 with *xylFGH* and *aphI* kanamycin resistance gene integrated at *xyl* providing sequences for homologous recombination of pXT10 and its derivatives; Km^r^	8
NT/v	NT127 *xylA^Δ4-804^*^::^*^tetAR^*; empty pXT10 cloning vector at the *xyl* locus; Tet^r^	8
NTlacZ	NT127 *xylA^Δ4-804^*^::^*^lacZ^*; *lacZ* coding sequence expressed via the *xylA* promoter replacing *xylA*; Tet^r^	8
NTΔcopZA	NTX Δ*copZA*::*aacC1*; Gm^r^ Km^r^	This study
NTΔcopZA/v	NTΔcopZA with empty vector pXT10 at the *xyl* locus; Gm^r^ Tet^r^	This study
NTΔcopZA/c	NTΔcopZA with *copZA* expressed under the endogenous promoter for complementation at the *xyl* locus; Gm^r^ Tet^r^	This study
NTΔcopA	NTX Δ*copA*::*aacC1*; Gm^r^ Km^r^	This study
NTΔcopA/v	NTΔcopA with empty vector pXT10 at the *xyl* locus; Tet^r^	This study
NTΔcopA/c	NTΔcopA with *copA* expressed under the endogenous promoter for complementation at the *xyl* locus; Gm^r^ Tet^r^	This study
NTΔcopZ	NTX Δ*copZ*::*aacC1*; Gm^r^ Km^r^	This study
NTΔcopZ/v	NTΔcopZ with empty vector pXT10 at the *xyl* locus; Gm^r^ Tet^r^	This study
NTΔcopZ/c	NTΔcopZ with *copZ* expressed under the endogenous promoter for complementation at the *xyl* locus; Gm^r^ Tet^r^	This study
NTΔcueR	NTX Δ*cueR*::*aacC1*; Gm^r^ Km^r^	This study
NTΔcueR/v	NTΔcueR with empty vector pXT10 at the *xyl* locus; Gm^r^ Tet^r^	This study
NTΔcueR/c	NTΔcueR with *cueR* expressed under the endogenous promoter for complementation at the *xyl* locus; Gm^r^ Tet^r^	This study
NTPcop-LacZ	NT/v P*copZ*::*lacZ* at the *xyl* locus; Km^r^	This study
NTΔcueRPcop-LacZ	NTΔcueR/v P*copZ*::*lacZ* at the *xyl* locus; Gm^r^ Km^r^	This study
Plasmids		
pXT10	Delivery vector for chromosomal expression at the *xyl* locus of H. influenzae, contains *xylF*, *xylB*, *xylA^Δ4-802^*, and *tetAR* Tet^r^ tetracycline resistance cassette; Tet^r^; referred to as v	49
pXRcopZA	pXT10 with RdAW *copZA* (6 copies of *copZ*) expressed under the endogenous promoter; Tet^r^	This study
pXNTcopZA	pXT10 with NT127 *copZA* (3 copies of *copZ*) expressed under the endogenous promoter; Tet^r^	This study
pXNTcopA	pXT10 with NT127 *copA* expressed under the endogenous promoter; Tet^r^	This study
pXNTcopZ	pXT10 with a single copy of NT127 *copZ* expressed under the endogenous promoter; Tet^r^	This study
pXNTcueR	pXT10 with NT127 *cueR* expressed under the endogenous promoter; Tet^r^	This study
pXKZ	Derivative of pXT10 containing *lacZ* and the *aphI* resistance gene; Km^r^	This study
pXTtrcKan	Derivative of pXT10 containing E. coli *trc* promoter driving the *aphI* resistance gene; Km^r^	This study

**FIG 3:**
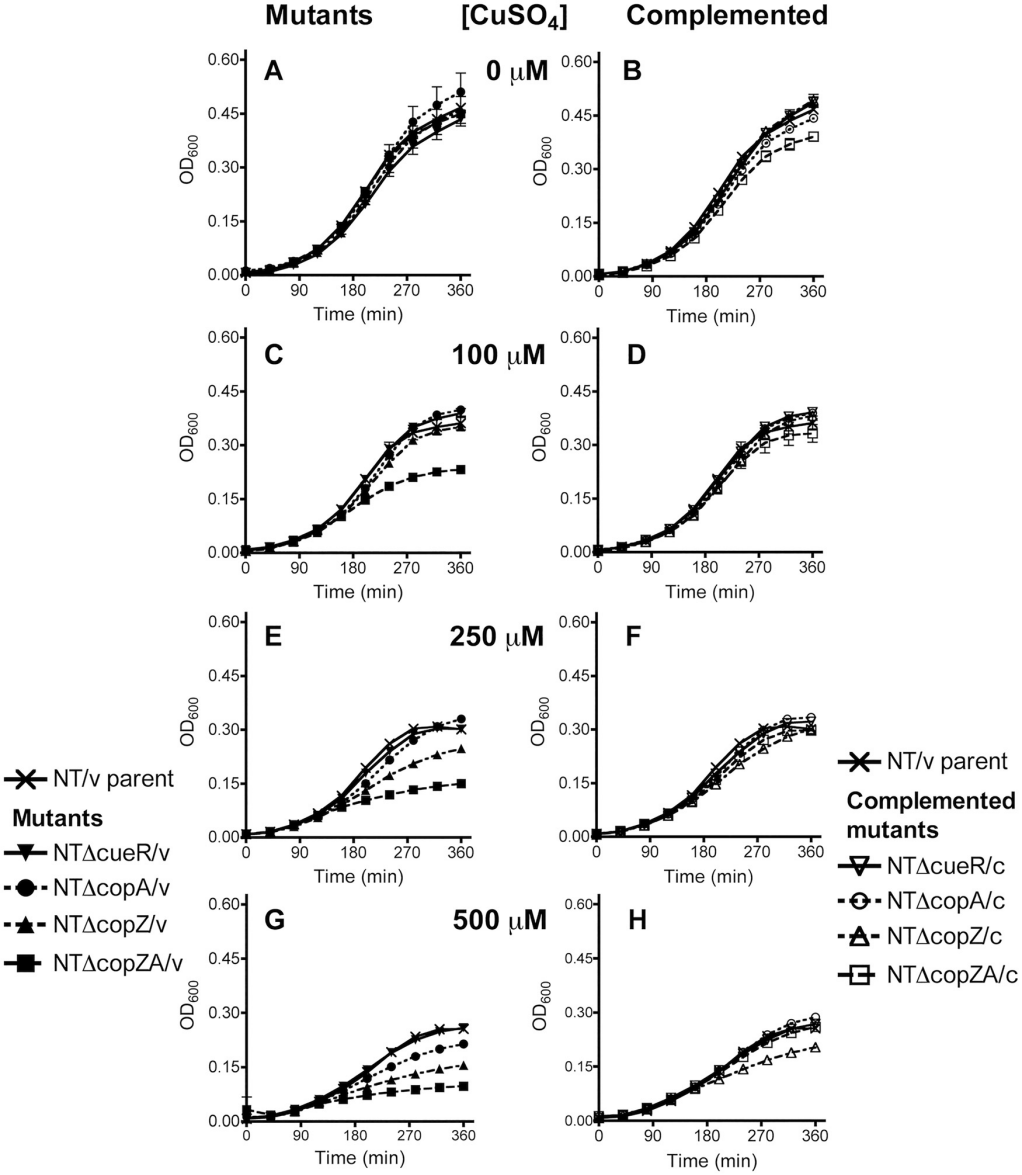
Role of *cueR*/*copZA* in copper resistance of NTHi. (A to H) Strains were grown aerobically in sBHI containing (A and B) no copper addition, (C and D) 100 μM CuSO_4_, (E and F) 250 μM CuSO_4_, and (G and H) 500 μM CuSO_4_ in 96-well plates. The graphs in the left column show the growth curves of the mutant strains (closed symbols), and those in the right column show the complemented strains (open symbols); the parental NT/v (×) growth data are included in both columns at each concentration for comparison to mutant and complemented strains. The results represent the mean and SD from two independent experiments.

To address the role of copper tolerance genes in a clinical isolate of NTHi, a series of defined mutants was constructed in NT127 by deletion of genes encoding the regulator (NTΔcueR/v), the chaperones (NTΔcopZ/v), the Cu^+^-ATPase (NTΔcopA/v), or both the chaperones and Cu^+^-ATPase (NTΔcopZA/v) (Table 1). Nonpolar deletions were constructed by replacing the coding regions with an antibiotic resistance marker, and mutants were complemented by expression of the intact genes ectopically at the *xyl* locus (Materials and Methods) (49). In growth assays comparing the effect of copper on NTHi strains, NTΔcopZA/v showed the greatest sensitivity to increasing copper concentrations, and the growth profile of this mutant deviated from other strains at concentrations equal to or greater than 100 μM CuSO_4_ (Fig. 3C). The strain NTΔcopZ/v displayed inhibition at 250 μM CuSO_4_ (Fig. 3E), while NTΔcopA/v displayed inhibition only after the addition of 500 μM CuSO_4_ (Fig. 3G). NTΔcueR/v grew similarly to the parental strain at all concentrations tested. Complementation restored copper tolerance to parental levels in strains containing the Δ*copZA*, Δ*copZ*, or Δ*copA* deletions (Fig. 3D, F, and H). However, NTΔcopZ/c displayed partial complementation (Fig. 3H), likely due to the presence of only one *copZ* gene in the complementing construct relative to the three copies in the parental strain. Together, these data suggest that the *copA* and *copZ* genes in H. influenzae have roles in copper resistance and likely contribute to additive effects observed for the Δ*copZA* phenotype.

### Regulation of the copper locus and analysis of promoters.

In the *copA* promoter of E. coli, the –35 and –10 motifs display extended 19-bp spacing and flank operator sites, a characteristic of MerR-type promoters. Metal-responsive regulators in this family can mediate repression and activation through modulation of DNA structure at the promoter site (50–53). Transcriptional activation of CueR occurs following a conformational DNA change induced by the regulator in the presence of Cu^+^ (41, 50, 54). The H. influenzae *cop* (i.e., *copZ1*) promoter contains features common to E. coli P*copA* (Fig. 1). In H. influenzae, the sequence of the apparent CueR binding site (arrows in Fig. 1) is an imperfect palindrome interrupted by seven base pairs, ACCTTaA-N_7_-TaAAGGT. This motif is conserved with 100% nucleotide sequence identity in 101/102 complete genome sequences of H. influenzae strains (NCBI database https://www.ncbi.nlm.nih.gov/genome/) and one strain showing 86% nucleotide sequence identity. When compared to the P*copA* of E. coli (ACCTTCC-N_7_-GGAAGGT), the motif differs at the last two nucleotides of the palindrome.

In H. influenzae genomes, *cueR* is divergently transcribed from the *copZA* operon. Thus, two promoters (P*cueR* and P*cop*) are likely located in this short intergenic region. A putative P*cueR* is predicted with 17-bp spacing between the –10 and –35 elements (bottom strand) (Fig. 1). In this proposed scheme, the –10 element of P*cueR* overlaps with the CueR box (right arrow) in the *cop* promoter, and this constraint may account for the differences between the CueR binding sequences of E. coli and H. influenzae.

**FIG 4:**
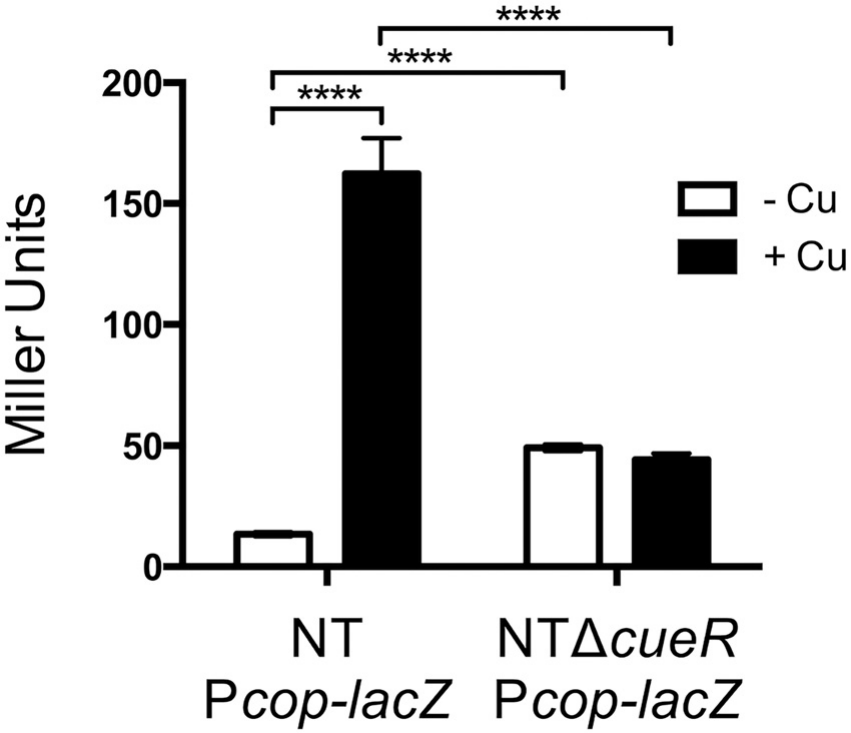
Effect of *cueR* deletion on expression of P*cop-lacZ.* β-Galactosidase activity of the transcriptional reporter in parental and Δ*cueR* backgrounds were monitored from cultures grown in sBHI in the absence or presence of 250 μM CuSO_4_. The data represent the averages of six independent replicates, and error bars denote the SD (****, *P < *0.0001).

To examine the activity of the *cop* promoter in H. influenzae in response to copper, a reporter strain, NTPcop-lacZ, was constructed (Table 1). This strain carries the 76-bp region between the initiation codons of *cueR* and *copZ1* (Fig. 1) fused to the promoterless *lacZ* gene of E. coli. Growth in the absence of copper addition resulted in minimal detection of β-galactosidase activity, while in the presence of 250 μM copper, the activity increased approximately 12-fold (*P < *0.0001) (Fig. 4). To determine if CueR regulates expression of the *cop* promoter in NTHi, the *Pcop-lacZ* fusion was integrated into a *cueR*-deficient strain, NTΔcueRPcop-lacZ. In this strain background, deletion of *cueR* abolished the copper-mediated induction of the *lacZ* fusion (Fig. 4). These findings are consistent with a role for CueR as a copper-responsive activator that senses and responds to cytoplasmic copper status as seen in other organisms (41, 54–56).

CueR_HI_ also mediates repression in the absence of toxic levels of copper, as ~3.7-fold higher levels of *lacZ* reporter expression were observed under this condition in the *cueR*-deficient strain compared to the parental *cueR*^+^ strain, NTPcop-lacZ (Fig. 4). The role of CueR as a repressor is also consistent with reports in E. coli studies that show its ability to switch from repressor to activator mode via a DNA-distortion mechanism in response to metal binding (57), as well as by modulating RNA polymerase interactions to either repress or activate transcription at the *copA* promoter (58).

### Deletion mutants of *copZA* accumulate copper.

**FIG 5:**
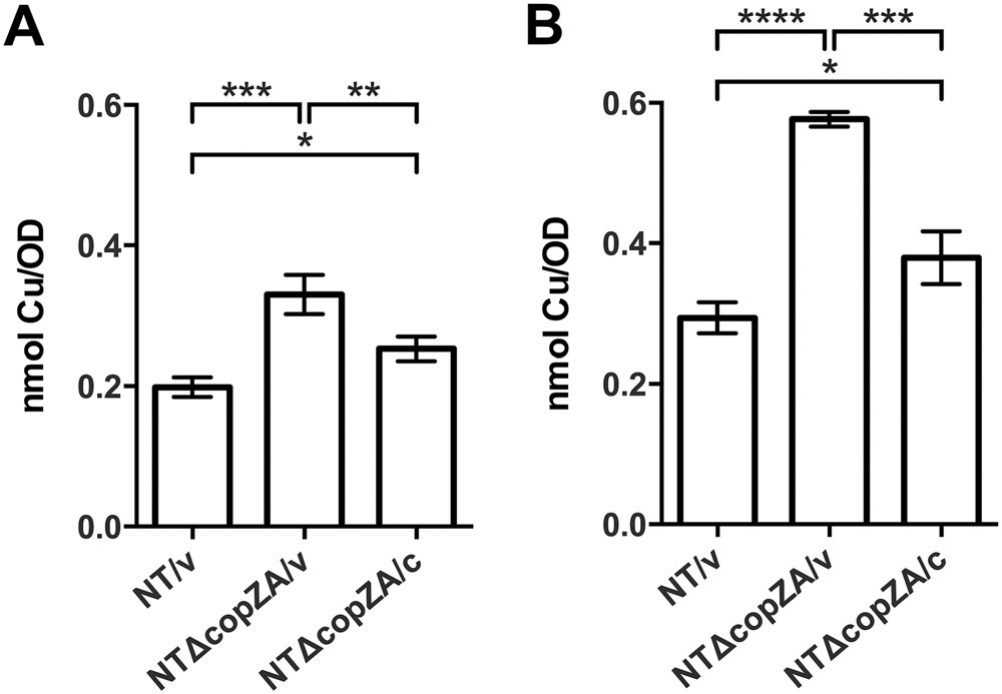
Analysis of cellular copper accumulation in NTHi. (A and B) Cultures were grown for 2 h in media containing (A) 250 μM and (B) 500 μM CuSO_4_. The copper content of NT/v, NTΔcopZA/v, and NTΔcopZA/c cells was measured by furnace AAS and normalized to the optical density of the sample. The results represent the mean of 3 independent replicates, and error bars denote the SD (*, *P < *0.05; **, *P < *0.01; ***, *P < *0.001; ****, *P < *0.0001).

Copper sensitivity of the *cop* mutants in H. influenzae suggests excess copper is not adequately removed and could potentially accumulate to cause toxicity. To examine cellular copper accumulation, strains were grown in media containing sublethal concentrations of copper, and the total copper levels were measured using furnace atomic absorbance spectrometry. Figure 5 shows that the NTΔcopZA/v mutant accumulated ~66% and ~97% more copper than parental strain NT/v when grown for 2 h in the presence of 250 μM (Fig. 5A) and 500 μM copper (Fig. 5B), respectively. Complementation of the mutation in NTΔcopZA/c restored its ability to lower cellular copper levels despite slightly elevated levels of 27% and 30% above those of NT/v at 250 μM and 500 μM, respectively.

### The *copZA* operon is required for NTHi infection of the mouse lung.

**FIG 6:**
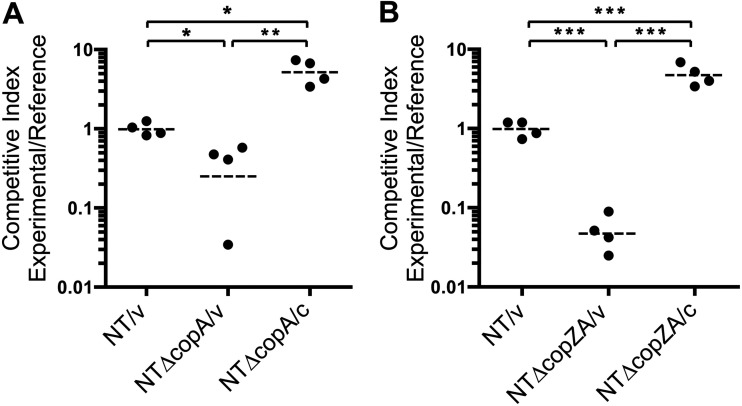
*copA* and *copZA* deletions decrease the fitness of NTHi in the lung. Mice were coinfected with the experimental and LacZ+ reference strain at a 1:1 ratio, and at 24 h postinfection CFU were enumerated from lung homogenates on indicator plates. Competitive indices were calculated by dividing the ratio of experimental strain CFU to reference strain CFU recovered from the infection and then normalized to that of the inoculum. The symbols represent individual animals, and dashed lines denote the geometric mean. (A and B) Mean CI values were (A) 0.987 for NT/v, 0.249 for NTΔcopA/v, and 5.19 for NTΔcopA/c and (B) 0.979 for NT/v, 0.047 for NTΔcopZA/v, and 4.70 for NTΔcopZA/c (*, *P < *0.05; **, *P < *0.01; ***, *P < *0.001).

Copper tolerance has been recognized as an important virulence mechanism in several pathogenic bacteria but not in all cases as mentioned earlier (20–32). To evaluate the fitness defect of the *copA*-deficient mutant predicted by HITS (Fig. S1) (6), NTHi strains were assessed for their ability to infect the lungs of 6-week-old C57BL/6 mice (Fig. 6). As duplicated genes such as *copZ* are functionally redundant and disruption of one copy does not result in any detectable phenotypic changes, this class of genes was excluded from our previous HITS analysis. To identify potential synergistic contributions of *copA* and *copZ* to survival *in vivo*, we also tested the *copZA* deletion mutant and its complemented derivative. The infections were conducted as competition experiments between an experimental strain and the *lacZ*-containing reference strain, NTlacZ (Table 1). At 24 h postinfection, competitive indices (CI) were calculated from CFU differentially enumerated on X-Gal (5-bromo-4-chloro-3-indolyl β-d-galactopyranoside)-containing media (Materials and Methods). Relative to the CI of the parent strain NT/v, mutations in *copA* and *copZA* (strains NTΔcopA/v and NTΔcopZA/v) were underrepresented during infection by ~4-fold (*P < *0.05) and ~20-fold (*P < *0.001) (Fig. 6A and B), respectively. This suggests that the *copZA* mutation is more attenuating than the *copA* mutation alone. Complementation restored the ability of these mutants to survive in the lung. CI ratios between NTΔcopA/c and NTΔcopA/v differed by ~20-fold (*P < *0.01), while NTΔcopZA/c and NTΔcopZA/v showed an ~100-fold difference (*P < *0.001) (Fig. 6A and B, respectively), indicating that the attenuation in virulence was specific to the mutations of *copA* and *copZA*. In fact, the complemented strains of the respective mutants exhibited slight competitive advantages relative to the parent strain, as comparisons of CI ratios between each of the NTΔcopA/c and NTΔcopZA/c groups versus NT/v showed an ~5-fold difference (*P < *0.05 and *P < *0.001, respectively). It is possible that *in vivo* expression of the complementing genes at the *xyl* locus exceeds that of the parental strain, leading to improved survival. Overall, the results from the lung infection data indicate that the copper efflux system is required for colonization and survival of pathogenic NTHI in the mouse lung.

## DISCUSSION

To tolerate excess copper and prevent its toxic effects, bacteria use multiple pathways to maintain copper homeostasis in the cytoplasm and additionally in the periplasm of gram-negative bacteria. The mechanisms of copper transport and homeostasis have been best characterized in prototypical model species such as Escherichia coli and Pseudomonas syringae for gram-negative bacteria and Enterococcus hirae for gram-positive bacteria (9–11). One major copper transport system encoded by the *cue* (Cu efflux) locus (first identified in E. coli) encodes a copper-responsive regulator CueR, a periplasmic multicopper oxidase CueO, and a P-type ATPase transporter CopA responsible for controlling cytosolic copper levels (41, 44, 54, 55). Subsequently, it was determined that the *copA* gene of E. coli also encoded a second protein, CopZ, a copper chaperone expressed as a result of programmed ribosomal frameshifting (59, 60). The *cus* system (*cusCFBA*), also first identified in E. coli (61), is proposed to remove excess levels of copper from the periplasmic to extracellular space (9, 10). In addition to these chromosomally encoded copper resistance genes, the Pco systems are plasmid-borne copper resistance determinants first characterized in E. coli (*pcoABCDRSE*) and Pseudomonas syringae (*copABCDRS*) with *cop* homologs identified in other pseudomonads (9, 10).

Most bacterial genomes encode at least one copper efflux system where the Cu-ATPase (*copA*) is usually present in an operon with a Cu-responsive transcriptional regulator and a copper metallochaperone. Typically, the metallochaperone (usually called *copZ*) is encoded by a separate gene (62), unlike the programmed frameshift seen in E. coli. In most gram-negative bacteria, *copA* is activated by a CueR homolog, whereas in most gram-positive bacteria and Mycobacterium tuberculosis the Cu ATPase is negatively regulated by CsoR (e.g., Bacillus subtilis) (42) or CopY (e.g., Enterococcus hirae, Streptococcus spp.) (11, 23, 47, 63).

Gram-negative bacteria have homologs or variations of the Cue, Cus, or Pco (Cop) systems with homolog names differing between and sometimes within bacterial species. Some possess multiple copper resistance systems, such as E. coli and Pseudomonas spp. In this report, we showed that H. influenzae appears to rely primarily on a simplified copper defense system which comprises a copper-responsive regulator *cueR*, tandemly repeated copper chaperone genes, *copZ(n)*, and P-type Cu-ATPase *copA* organized adjacently in the genome (Fig. 1). We showed that NTHi single *copA*, *copZ*, and double deletion *copZA* mutants are defective for copper tolerance in growth toxicity assays (Fig. 3). Complementation of these mutants restored growth in excess copper, indicating that these gene products function to detoxify copper in this organism. The *copZA* gene products are specific for detoxifying excess copper and not cobalt, zinc, or manganese (Fig. 2). Moreover, deletion of *copZA* resulted in cellular copper accumulation in the presence of increasing amounts of CuSO_4_ during growth compared to the parent and complemented strains, consistent with the role of this system in copper transport and likely accounting for the growth inhibition (Fig. 5). Our collective results are consistent with observations that *cop* transport/chaperone mutants in diverse pathogenic bacteria are growth inhibited under excess copper conditions that lead to accumulated intracellular copper (20, 23, 25, 28, 30, 31, 47, 64, 65). For example, in Streptococcus mutans, *copYAZ* is required for copper resistance, and a *copYAZ* mutant accumulates a 2-fold increase in the amount of intracellular copper compared to the wild-type strain (47).

We also showed with a *cop* promoter-driven *lacZ* reporter fusion that expression of the *copZA* operon is activated by copper under the regulatory control of CueR, consistent with its function as the transcriptional regulator that senses intracellular copper and activates synthesis of the *copA* efflux pump (Fig. 4). CueR_Hi_ is within the MerR family of regulators first described in E. coli as a transcriptional activator of the Cu ATPase *copA* and copper efflux oxidase *cueO* genes (41, 54, 55). Promoter *lacZ* fusion assays in E. coli in these cited studies showed induction of the *copA* promoter by copper which was abolished in a mutant with a deletion of *cueR* (*cueR* is termed *copR* in the Petersen and Møller study [55]). In addition to its role as a transcriptional activator, CueR can also act as a repressor in the absence of copper (57), which we observed for NTHi in this report. It is not clear why negative regulation by CueR of *cop* promoter *lacZ* fusions in the absence of copper has been seen in some studies (56), including ours here, and not in others (41, 54, 55), but perhaps CueR repressors have alternative regulatory properties in different bacteria.

We verified our previous HITS screening data indicating that a mutation in *copA* attenuates survival in the mouse lung (6, 7). We also showed a contribution of the chaperone genes in the *copZA* mutant for *in vivo* survival after deletion of tandem copies of *copZ* compared with deletion of *copA* alone. The competitive indices suggest that both NTHi *copZA* and *copA* mutants were attenuated (~20- and 4-fold, respectively) compared to the parental strain, and complementation of these mutants restored survival above parental levels, indicating that the attenuation was specific to these mutations (Fig. 6). Deletion of *copA* and the duplicated *copZ* genes together caused greater attenuation in mouse lungs than deleting *copA* alone, establishing a contributory role for *copZ* and indicating that the copper chaperones and efflux ATPase are needed together for optimal survival *in vivo*. These results correlate with the more severe growth inhibition seen with the *copZA* double mutant versus the *copA* single mutant in the presence of increasing concentrations of CuSO_4_ (Fig. 3), indicating synergism of the *copZ* and *copA* gene products for *in vitro* copper tolerance and *in vivo* pathogenicity. The additive effect of deleting *copZ* in the *copA* background suggests that *copZ* mediates a *copA*-independent function in copper resistance. Future studies will be required to determine whether CopZ interacts with an additional transporter or possesses a copper-sequestering capability.

The *copA* gene plays an important role in mouse pulmonary infection by several bacterial species; however, this is not always the case, and the role of *copZ* in pathogenesis has been underexamined. In animal models *copA* mutants have shown attenuation ranging from ~10-fold to 2-log differences compared to parent strains at 24 h of infection for the respiratory pathogen Streptococcus pneumoniae (22, 23) and opportunistic pathogens such as Klebsiella pneumoniae (21) and Acinetobacter baumannii (20). However, a *copA* mutant was not attenuated for lung colonization compared to the parent in the porcine respiratory pathogen Actinobacillus pleuropneumoniae (31). In infection studies with M. tuberculosis, a mutant lacking the *copA*-like P-type ATPase exhibited a bacterial burden equivalent to that of the parental strain but less lung disease severity in mice (and guinea pigs) (66). In contrast, effective lung (and lymph node) colonization by M. tuberculosis required a separate putative copper transporter in the guinea pig model (67). Recently, in Bordetella pertussis, deletion of a three-gene operon encoding CopZ, glutathione-dependent peroxidase, and glutathione reductase did not affect colonization appreciably in mouse lungs, yet deletion of this operon did cause a reduction in persistence in the nasal cavity, suggesting that CopZ may contribute to nasopharyngeal colonization in this organism (45).

One interesting feature of the H. influenzae *cop* system is the tandem duplication of the metallochaperone gene, *copZ*. The adjacent genomic organization of *cueR*, *copZ*, and *copA* appears to be conserved in all H. influenzae strains and other members of the *Pasteurellaceae* family; however, we found that tandem variable duplication of *copZ* appears to be unique to H. influenzae and its close relative Haemophilus haemolyticus and to our knowledge has not been reported in other bacterial systems. Pseudomonas aeruginosa has been reported to have two copper chaperones, CopZ1 and CopZ2, but they have distinct functions, sharing only 37% amino acid sequence identity to each other, and are located in different genomic locations (68, 69). Maintenance of gene duplication in bacteria is thought to be an adaptation to environmental stress to survive in diverse environments (70–73). Perhaps this is the case for maintaining multiple copies of the copper chaperone gene in H. influenzae. Gene duplication of virulence determinants such as capsule and pilus have been documented in H. influenzae and appears to correlate with increased pathogenicity (74). The *cap* locus (11 genes encoding capsule production) has 10 of these 11 genes duplicated in a cluster in H. influenzae type b (75–77), whereas in H. influenzae biogroup *aegyptius*, the entire *hifABCDE* locus encoding pilus production is duplicated (78, 79). During the course of genome sequencing and assembly of several H. influenzae biogroup *aegyptius* strains, Phillips et al. (80) noticed that the genome size of one of the strains sequenced was ~28 kb larger than the whole-genome shotgun contigs of the same strain deposited by another group. The authors concluded that the discovery of extra sequences was due to using a long-read sequencing platform instead of short-read sequencing. Interestingly, the extra sequences are duplications of several regions including known virulence determinants such as genes that encode for lipooligosaccharide (LOS) phosphorylcholine addition, pilin, and pilus export.

Gene duplication as an adaptive response to metal resistance has been documented in a variety of bacteria and fungi (70, 81). For example, copper sequestration studies in the opportunistic pathogen Ralstonia pickettii showed that when isolated from lake sediment contaminated with high levels of copper, it had adapted by duplicating metal resistance and transporter operons, although tandem duplication of the copper chaperone gene was not reported (82). In the budding yeast Saccharomyces cerevisiae, adaptive gene amplification of the copper binding metallothionein gene CUP1 is associated with copper tolerance and detoxification (83). The mechanism by which *copZ* duplications have occurred in H. influenzae is unknown, but gene duplication as a defensive strategy likely favors survival against copper stress in host environments.

Does H. influenzae have multiple copper efflux systems? First, with respect to the E. coli Cue system, H. influenzae does not appear to encode the multicopper oxidase CueO in its genome. A BLASTP (84) homolog search with E. coli CueO identified a gene annotated as SufI (a member of the multicopper oxidase family) in H. influenzae. To our knowledge, no studies of SufI have been reported for this organism; however, the SufI (FtsP) homolog in E. coli was shown to not bind copper and functions specifically in cell division (85, 86). H. influenzae does not appear to have a *cus* copper/silver efflux system for transporting excess copper from the periplasm to the extracellular milieu. BLASTP (84) searches of the H. influenzae NCBI taxonomy database (87) against E. coli *cusCFBA* genes did not identify significant sequence similarities to CusF (periplasmic chaperone that transports copper in the periplasm to the pump encoded by *cusCBA*) and CusB (periplasmic membrane fusion protein). However, sequence similarities to CusC were identified (outer membrane channel-forming protein) and CusA (cytoplasmic membrane substrate binding and transport protein belonging to the resistance-nodulation-division [RND] family of efflux pumps). CusC has ~25% amino acid sequence identity to the outer membrane protein TolC in H. influenzae (HI1462) (88, 89), and CusA has ~23% amino acid sequence identity to multidrug efflux RND transporter permease subunit AcrB in H. influenzae (HI0895) (90–93). AcrB and TolC are components of the tripartite AcrAB-TolC efflux pump that contribute to multidrug resistance found in gram-negative bacteria (94), including H. influenzae (89).

Studies of E. coli have shown that TolC is very similar structurally to CusC (95) but cannot functionally replace CusC (96). Interestingly, several recent reports have shown TolC to have a role in copper tolerance. In the nitrogen-fixing bacterium Bradyrhizobium liaoningense, a transposon mutant library grown under copper selection identified *tolC* among six other genes to be involved in copper resistance (97). The *tolC* gene in the cyanobacterium *Synechocystis* has a role in copper efflux, as growth of a *tolC* mutant is inhibited greater than that of the wild type in the presence of copper and also accumulated ~3-fold more intracellular copper than the wild type (98). Also, a chemical genetic screen of the Keio collection of 3,985 E. coli deletion mutants with prolonged exposure to copper identified *tolC* as required to tolerate copper stress (99). Together, these studies suggest the possibility that an unidentified TolC-dependent efflux system analogous to the *cus* system (which utilizes a TolC-like protein) may be involved in copper resistance, and it remains to be seen if such a system may represent the periplasmic copper efflux system of H. influenzae.

Copper plays an important role at the host pathogen interface, as it is used by cells of the innate immune system as an antimicrobial agent (19, 100). Copper transport into macrophages has been shown to promote bactericidal activity, and bacterial systems such as E. coli, *S.* Typhimurium, and S. pneumoniae containing deletions in copper exporters (e.g., CopA, GolT) were more susceptible to macrophage-mediated killing (17, 22, 26). It would be interesting in future studies to examine the H. influenzae Cu^+^-ATPase and chaperone mutants for susceptibility to killing by macrophages. Another interesting attribute associated with the copper efflux system is its role in biofilm formation, as growth in a biofilm mode is used by many bacteria to survive in response to environmental stresses such as nutrient limitation, pH and temperature changes, and exposure to antimicrobial agents and, as such, may have a role in the pathogenesis of disease (101). For example, in S. pyogenes, which causes a wide range of clinical illnesses, biofilm formation of the wild-type isolate was inhibited by copper (63), while CopZ in the dental-associated pathogens Streptococcus mutans and Streptococcus gordonii was critical for biofilm formation and detachment, respectively, *in vitro* (102, 103). It remains to be seen whether the *cop* system of H. influenzae might also have a role in biofilm formation/detachment.

In summary, our findings in this report increase our understanding of NTHi pathogenesis, as copper is a host immune defense used to intoxicate invading pathogens. As recent work has demonstrated that a copper efflux system can be targeted with antibacterial oligopeptides that enhance copper-mediated toxicity (104), understanding copper tolerance pathways in NTHi could help in designing potential antimicrobial strategies targeting copper homeostasis in this respiratory pathogen of significant clinical relevance.

## MATERIALS AND METHODS

### Strain and culture conditions.

Haemophilus influenzae RdAW and Rd KW20 nonencapsulated serotype d derivatives, NTHi strain NT127, and all other H. influenzae strains listed in Table 1 were grown in brain heart infusion (BHI) broth supplemented with 10 μg/mL hemin and 10 μg/mL NAD (sBHI) or on sBHI agar plates at 35°C. When necessary, antibiotics were added to media at the following concentrations: 8 μg/mL tetracycline (Tc), 20 μg/mL kanamycin (Km), and 10 μg/mL gentamicin (Gm). Rd KW20 is the complete genome sequenced reference strain (105), and based on the annotation in KEGG (https://www.genome.jp/kegg/), we use the following gene designations: Rd KW20 locus tag HI0293, *cueR*; HI0292, HI0291, *copZ* genes, and HI0290, *copA*. The draft genome sequences of RdAW and NTHi NT127 are deposited in contigs available under GenBank accession no. ACSM00000000 (consists of sequences ACSM01000001 to ACSM01000032) and accession no. ACSL00000000 (consists of sequences ACSL01000001 to ACSL01000041), respectively. The locus tag numbers for the corresponding *cueR*, *copA*, and *copZ* genes in RdAW and NT127 with their corresponding accession numbers are illustrated in Fig. S2. In RdAW, we termed the locus tag HICG_01412, *cueR*; HICG_01413, HICG_01718 HICG_01717, HICG_01008, *copZ* (n) genes; and HICG_01009, *copA*. In NT127, we termed the locus tag HIAG_01035, *cueR*; HIAG_01036, HIAG_01809, HIAG_01773, *copZ* (n) genes; and HIAG_01772, *copA*. For simplicity, RdAW is termed Rd when describing mutant construction for this strain (below).

### Plasmid and strain construction.

Standard molecular biology techniques (106) were used for PCR, cloning, and plasmid construction. Nonpolar deletions of genes or regions of interest were constructed via gene replacement with the *aacCI* Gm-resistance (Gm^r^) cassette, which comprises 228 bp of sequence upstream of the initiation codon and the complete aminoglycoside-(3)-acetyltransferase coding sequence. The Gm^r^ cassette was fused to flanking H. influenzae fragments by overlap extension PCR (OE-PCR) (107) via tails added to the amplification primers. PCR products used as templates in splicing reactions were gel purified (Qiagen). The primers used in the study are listed in Table S1. For complementation of H. influenzae mutants, DNA fragments were amplified by PCR and cloned between adjacent restriction sites of the chromosomal delivery vector pXT10, as previously described (49). Typically, pXT10 was digested with SapI or the isoschizomer BspQI, and inserts were digested with EarI or BspQI. Ligation reactions were dialyzed and electroporated into E. coli DH5α. Following purification, plasmids were linearized by digestion with ApaLI prior to transformation and selection for double crossover homologous recombination. Competent cell preparation and transformation was accomplished as previously described (108). The plasmids used in the study are listed in Table 1.

### Construction of H. influenzae Rd mutants.

The Rd RdΔcopZA mutant was constructed by replacement of the region containing the six metallochaperones (*copZ*_1–6_) and HICG_01009 (*copA*) coding sequences with the Gm^r^ cassette. The replacement construct was created from three fragments: a 5′ flanking fragment (697 bp) from Rd, amplified using primers 5CuATPase1 and 3CuATPase1pG; the Gm^r^ cassette (762 bp), using primers 5pGent1 and 3Gent2, and a 3′ flanking region (796 bp) from Rd, using primers 5CuATPase2pG and 3CuATPase2. Products were joined by OE-PCR using primers 5CuATPase1 and 3CuATPase2. The resulting 2,207-bp amplicon was added to competent cells of strain Rd and plated on sBHI agar containing Gm, and Gm-resistant (Gm^r^) transformants were isolated and verified by PCR. To generate the strain RdΔcopZA/v, which contains an empty complementation vector, RdΔcopZA was transformed with linearized pXT10, and Tc^r^ transformants were isolated and verified by PCR. For complementation of the RdΔcopZA mutant, a segment containing the upstream promoter element, the six metallochaperones, and the ATPase gene was amplified by PCR with primers CuATP_comp_F and CuATP_comp_R, which bear restriction sites at the 5′ primer termini. The resulting 3,848-bp fragment was cloned into pXT10, creating plasmid pXRcopZA. This plasmid was linearized and added to competent cells of strain RdΔcopZA, and Tc^r^ recombinants were isolated and verified by PCR to yield strain RdΔcopZA/c.

### Construction of NTHi NT127 mutants.

To create strain NTΔcopZA, *copA* and the three *copZ* genes were deleted by transformation of the Gm-marked 2,207-bp stitched product from Rd (above) into strain NTX, a derivative of NT127 in which the xylose locus was modified for efficient recombination with pXT10-based plasmids (8). To generate NTΔcopZA/v, which carries the empty complementation vector, NTΔcopZA was transformed with linearized pXT10, and Tc^r^ transformants were isolated and verified by PCR. To complement the NTΔcopZA mutant, a segment containing the upstream promoter region, the three copper metallochaperones and the ATPase gene were amplified by PCR from NT127 with primers CuATP_comp_F and CuATP_comp_R. The resulting 3,006-bp fragment was cloned into pXT10 to create pXNTcopZA. This plasmid was linearized and added to competent cells of strain NTΔcopZA, and Tc^r^ recombinants were isolated and verified to yield strain NTΔcopZA/c.

The NTΔcopA deletion strain was constructed via gene replacement of *copA* (HIAG_01772) through the splicing of two fragments: the 5′ flanking product, amplified using primers 5CuATPase1 and 3HI0290_1pG from NT127 cells as template, and a product containing the Gm^r^ cassette and 3′ flanking region, amplified using 5pGent1 and 3CuATPase2 from NTΔcopZA cells as template. The intact replacement construct, amplified using primers 5CuATPase1 and 3CuATPase2, was transformed into NTX. Following selection on sBHI-Gm plates, isolates were validated by PCR to yield NTΔcopA, which was then transformed with linearized pXT10 to create NTΔcopA/v. To complement the *copA* deletion, two fragments were amplified from the pXNTcopZA plasmid template (a 1,168-bp fragment PCR amplified using primers x-xylF and HI0290_comp_R and a 2,180-bp fragment generated using primers HI0290_comp_F and CuATP_comp_R) and spliced by OE-PCR using primer x-xylF and CuATP_comp_R to generate a 3,327-bp product. From this intermediate, primers CuATP_comp_F and CuATP_comp_R were used to amplify a 2,239-bp fragment, which was cloned into pXT10 to create pXNTcopA. This plasmid was linearized and transformed into NTΔcopA to generate strain NTΔcopA/c.

To delete the three chaperone genes, a replacement construct was created from two fragments: a 1,459-bp fragment, amplified using primers 5CuATPase1 and 3gent2_290tail from NTΔcopZA cells as template, and a 987-bp fragment, amplified with primers HI0290_comp_F and q3ATPase from NT127 cells as template. These fragments were joined by OE-PCR using primers 5CuATPase1 and q3ATPase, and the resulting 2,422-bp amplicon was transformed into competent NTX cells. Gm^r^ transformants for strain NTΔcopZ were verified by PCR. Strain NTΔcopZ was transformed with pXT10 and selected for Tc^r^, and isolates were validated by PCR to yield NTΔcopZ/v. To complement the Δ*copZ* mutation, a single *copZ* gene was amplified using primers CuATP_comp_F and Chap_comp_R, as attempts to clone all three chaperones alone were unsuccessful. This 302-bp amplicon was cloned into pXT10 to create pXNTcopZ. Linearized pXNTcopZ was transformed into NTΔcopZ to generate NTΔcopZ/c.

The NTΔcueR mutant strain was constructed by replacement of the *cueR* (HIAG_01035) coding sequence with the Gm^r^ cassette. Three segments were fused by OE-PCR: a 1,290-bp 5′ fragment amplified using primers 5cueR1 and 3cueR1pG from NT127 cells as template, the 762-bp Gm^r^ cassette amplified with 5pGent1 and 3Gent2, and the 672-bp 3′ fragment amplified using 5cueR2pG and 3cueR2 from NT127 template. The resulting 2,683-bp product was transformed into NTX and NT/v to yield NTΔcueR and NTΔcueR/v, respectively. For complementation of the *cueR* mutation, a fragment consisting of the upstream elements and the *cueR* coding sequence was amplified using primers cueR_comp_F and cueR_comp_R. The resulting 486-bp product was digested and cloned into pXT10 to create pXNTcueR, which was transformed in NTΔcueR to yield NTΔcueR/c.

### Construction of *cop* promoter LacZ reporter.

To monitor *cueR*-dependent regulation, the *copZ* promoter/operator region (between *cueR* and *copZ1*) was fused to the translational start of *lacZ* and cloned into the xylose locus. The reporter was constructed from the following: an 867-bp fragment amplified with primers x-xylF and 3xylFcop from pXT10 template, a 3,150-bp fragment amplified using 5copLacZ and lacZtrcKan from pXKZ template, and a 2,415-bp fragment amplified using primers IFTrcF and x-xylB2 from pXTtrcKan template. The 76-bp intergenic region between the divergently oriented *cueR* and *copZ1* genes was created via the overlapping primer tails 3xylFcop and 5copLacZ. The amplicons were spliced together via OE-PCR using primer XT10thyA-F and x-xylB2 to create a 6,404-bp final product. This product was transformed into strains NTΔcueR/v and NT/v and selected for Km-resistant colonies to create strains NTΔcueRPcopLacZ and NTPcop-LacZ, respectively. The *copZ* promoter region of the reporter fusion was verified in the resultant strains by sequencing in both directions using primers pXGPseqF and lac7.

### Sequencing of chaperone repeats.

H. influenzae strains RdAW and NT127 were previously sequenced as whole-genome shotgun sequencing projects with GenBank accession numbers NZ_ACSM00000000 and NZ_ACSL00000000, respectively. For gap closure of shotgun contigs containing the copper efflux operon, PCR with primers CuChapSeq_F and CuChapSeq_R was used in amplification and sequencing (Fig. S2). The length of the operon between *cueR* and *copA* in additional NTHi strains was surveyed by PCR. Cells from single colonies or genomic DNA were used as template in PCRs using primers q5MerR and 5merR1 (Fig. S3). Samples were analyzed by agarose gel electrophoresis and imaged using a Gel Logic 200 system (Kodak) followed by staining with ethidium bromide.

### Metal sensitivity and growth curve analysis.

Standing overnight cultures were inoculated by a 1/20 transfer into 5 mL of sBHI in culture tubes and incubated with shaking at 250 rpm and 35°C until mid-log phase. These cultures were adjusted to 0.09 optical density (OD)/mL, and 25 μL was inoculated into 200 μL sBHI medium in wells of a 96-well microtiter plate (Corning) to a final OD/mL of 0.01. For growth assays, sBHI was supplemented with metals (CuSO_4_, MnCl_2_, CoCl_2_, and ZnSO_4_) at final concentrations of 0.032, 0.064, 0.128, 0.256, 0.512, and 1.024 mM or not supplemented. Microtiter plates were incubated at 35°C for 16 h in a VersaMax microplate reader (Molecular Devices, Sunnyvale, CA), and absorbance was recorded at 600 nm every 6 min. Growth curves were analyzed in Microsoft Excel and GraphPad Prism software.

### Reporter expression analysis.

Strains were grown in duplicate or triplicate biological replicates in 5 mL sBHI with or without the CuSO_4_ (250 μM final concentration) in culture tubes following a 1/10 transfer of standing overnight culture into sBHI at 35°C with shaking at 250 rpm. Absorbances of 200-μL culture samples were read at 600 nm in a microtiter plate reader. The β-galactosidase assay was performed as reported (109) with slight modification. To wells of a polypropylene 96-well DeepWell plate (Nunc 260251) containing 750 μL Z buffer, 15 μL 0.1% SDS, and 30 μL chloroform, 200 μL culture was added, mixed vigorously, and allowed to phase separate. Next, 100 μL of the permeabilized cells was added to a microtiter plate containing 20 μL *o*-nitrophenyl-β-d-galactopyranoside (ONPG; 4 mg/mL) and mixed. Absorbance was read at 420 nm in a kinetic assay every min for 30 min.

### Murine lung infection model.

Standing overnight cultures were used to inoculate 25 mL of sBHI in a 50-mL flask to a final optical density at 600 nm (OD_600_) of 0.01. The resulting cultures were incubated with shaking at 250 rpm and 35°C to mid-log phase. Experimental strains were mixed with the NTlacZ reference strain at a 1:1 ratio, washed, and diluted in Hanks’ balanced salt solution to a final concentration of 5 × 10^8^ CFU/mL. Then, 40 μL of bacteria (2 × 10^7^ CFU total) was inoculated into the nares of female 6-week-old C57BL/6 mice (Charles River Laboratories, Boston, MA) anesthetized with ketamine (65 mg/kg of body weight) and xylazine (6.5 mg/kg) by intraperitoneal injection. At 24 h of infection, lungs were harvested, homogenized, and plated on sBHI agar plates with 1 mM d-xylose and X-Gal (5-bromo-4-chloro-3-indolyl β-d-galactopyranoside; Sigma-Aldrich, St. Louis, MO) for CFU enumeration. Ratios of CFU of the experimental strains (white colonies, LacZ–) to competitor strain (blue colonies, LacZ+) were reported as the competitive index. All animal procedures were conducted in accordance with NIH guidelines and with prior approval by the University of Massachusetts Medical School Institutional Animal Care and Use Committee.

### Copper accumulation.

Overnight cultures were used to seed fresh 10-mL cultures and were grown to mid-log phase. Cells were harvested and adjusted to 1 OD/mL, and 250 μL was used to inoculate 10 mL of sBHI in 50-mL DeLong flasks (0.025 OD/mL final). Cultures were grown for 45 min and then supplemented with 10.5 mM or 21 mM CuSO_4_ (diluted in sBHI) for a final concentration of 250 μM or 500 μM CuSO_4_, respectively. Cultures were grown at 35°C with shaking at 250 rpm for 45 min or 2 h. Cells were harvested by centrifugation and washed twice with 5 mL of 150 mM NaCl and 10 mM HEPES, pH 7.5. Cell pellets were digested with 0.5 mL of NO3H (trace metal grade) for 1 h at 80°C and further incubated overnight at room temperature. Digestions were terminated by the addition of 0.1 mL of 30% H_2_O_2_. Samples were diluted 1:5 with water, and copper content was measured by furnace atomic absorption spectroscopy (AAS) (Varian SpectrAA 880/GTA 100, Santa Clara, CA).

### Statistical analyses.

Statistical significance was determined by one-way analysis of variance (ANOVA) with Tukey’s (Fig. 4 and 5) or Newman-Keuls (Fig. 6) multiple-comparison test using GraphPad Prism software (San Diego, CA).
