# Supplementary material for: Copper Efflux System Required in Murine Lung Infection by Haemophilus influenzae Composed of a Canonical ATPase Gene and Tandem Chaperone Gene Copies
Source: Infect Immun. 2023 Apr 4;91(5):e00091-23. doi: 10.1128/iai.00091-23 (PMC10187127; doi:10.1128/iai.00091-23)
Supplement: Supplemental file 1 — Fig. S1 to S4 and Table S1. Download iai.00091-23-s0001.pdf, PDF file, 1.2 MB [file iai.00091-23-s0001.pdf]

Supplemental Figure 1

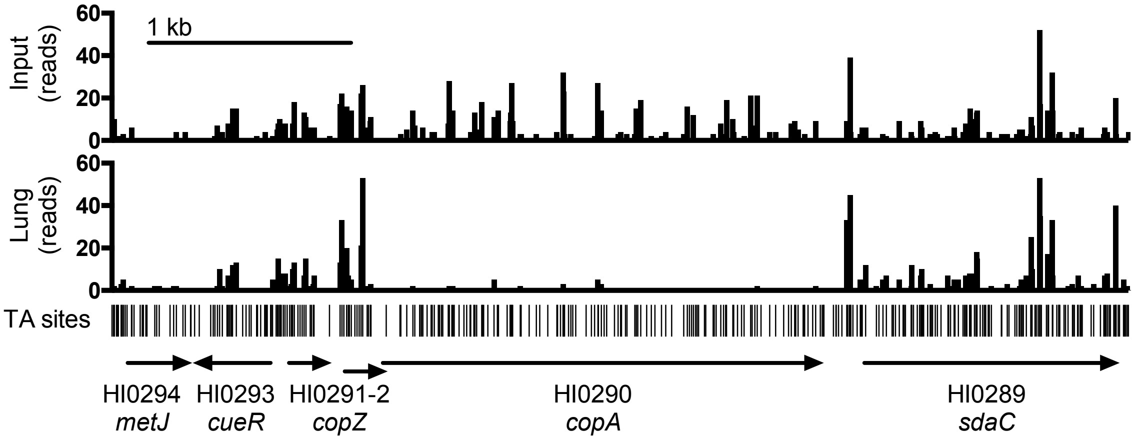

FIG. S1. HITS profile of the copper locus in *H. influenzae* before and after infection. Transposon insertion mutations were mapped to the *H. influenzae* Rd KW20 reference genome (NCBI database, Genbank accession no. L42023.1). The relative positions of the input and lung-selected libraries are displayed on the x-axis, and the bar height denotes the number of sequencing reads mapped to a particular insertion. Genes are indicated as arrows. Known TA dinucleotide sequences are displayed below the profiles. Data extracted from Gawronski et al. (1).

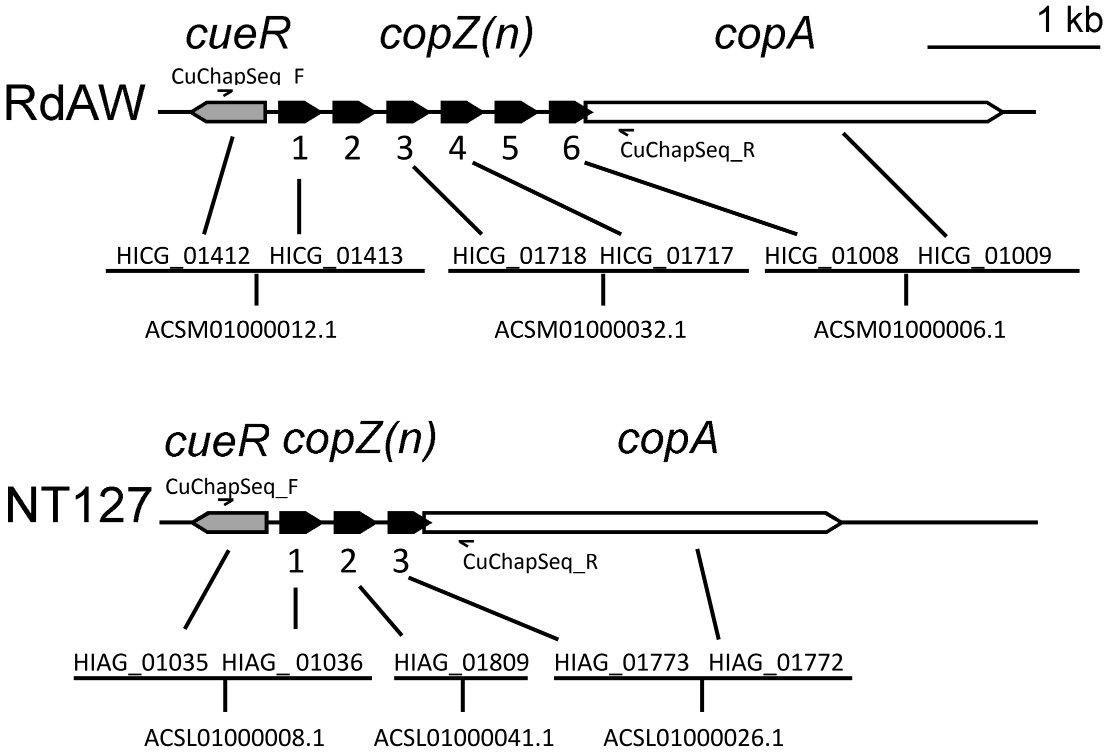

Fig. S2. *cop* region (*cueR*, *copZ (n)-copA*) in *H. influenzae* strains RdAW and NT127. RdAW is represented by locus tag nos. HICG_ , GenBank accession no. ACSM01000000 (consists of sequences ACSM01000001-ACSM01000032); NT127 is represented by locus tag nos. HIAG_ , GenBank accession no. ACSL01000000 (consists of sequences ACSL01000001-ACSL01000041). Gap closure of whole-genome shotgun contigs containing the copper efflux operon in RdAW and NT127 was completed via PCR amplification and sequencing with primers CuChapSeq_F and CuChapSeq_R (Table S1).

Supplemental Figure 3

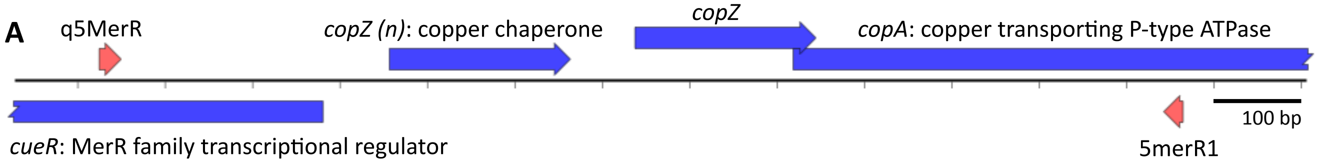

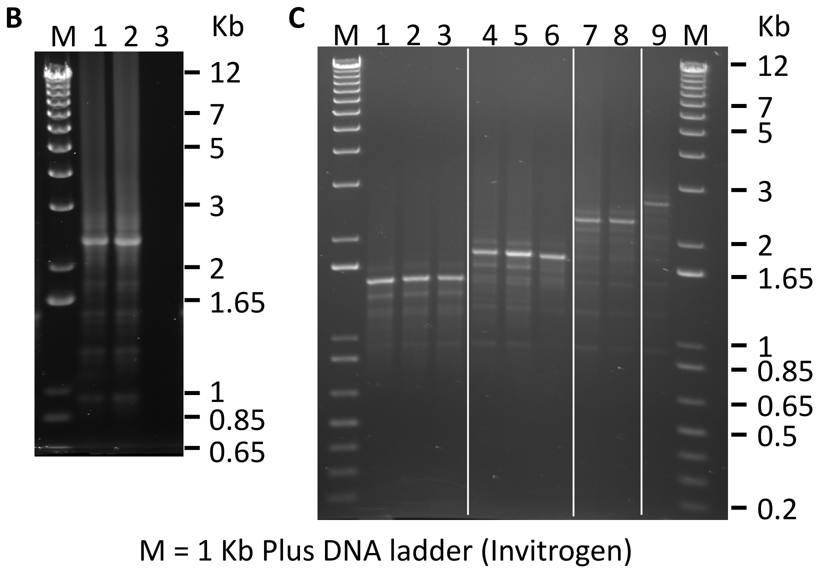

D

Lane	Strain	*copZ* copies	Size (bp)
panel B			
1	Rd KW20	6	2362
2	RdAW	6	2362
3	primers only		
panel C			
1	NT127	3	1519
2	PittII	3	1519
3	22.4.21	3	1519
4	PittEE	4	1800
5	PittGG	4	1800
6	22.1.21	4	1800
7	6P18HI	6	2362
8	RdAW	6	2362
9	86-028NP	7	2643

Fig. S3. *cop* region of *H. influenza*e. (**A**) Map illustrates variation in the copy number (n) of the *copZ* chaperone gene located between *cueR* and *copA* in *H. influenzae* strains. Length and number of *copZ* repeats predicted between *cueR* and *copA* determined by PCR using primers q5MerR and 5merR1 in Rd KW20 and RdAW (**B**) and amongst eight NTHi strains (**C**). (**D**) Lane designations for each gel.

CopZ amino acid sequence alignment

```
NT127copZ1   MKTITLNIKGIHCGCCVKSLTQVLTELDGVQSADVQLEGKVNITFDENRVNVAQLIEVIE   60
NT127copZ3   MKTITLNIKGIHCGCCVKSLTQVLTELDGVQSADVQLEGKVNITFDENRVNVAQLIEVIE   60
NT127copZ2   MKTITLNIKGIHCGCCVKSLTQVLTELDGVQSADVQLEGKANITFDENRVNVAQLIEVIE   60
RdAWcopZ1    MKTITLNIKGIHCGCCVKSLTQVLTELDGVQSADVQLEGKANITFDENRVNVAQLIEVIE   60
RdAWcopZ2    MKTITLNIKGIHCGCCVKNLTRVLTELDGVQSADVQLEGKANITFDENRVNVAQLIEVIE   60
RdAWcopZ3    MKTITLNIKGIHCGCCVKNLTQVLTELDGVQSADVQLEGKANITFDENRVNVAQLIEVIE   60
RdAWcopZ4    MKTITLNIKGIHCGCCVKNLTQVLTELDGVQSADVQLEGKANITFDENRVNVAQLIEVIE   60
RdAWcopZ5    MKTITLNIKGIHCGCCVKNLTQVLTELDGVQSADVQLEGKANITFDENRVNVAQLIEVIE   60
RdAWcopZ6    MKTITLNIKGIHCGCCVKNLTQVLTELDGVQSADVQLEGKANITFDENRVNVAQLIEVIE   60
             ******************.**:******************.*******************
```

Semi conserved | conserved | Semi conserved

```
NT127copZ1   DAGFDATE* 68
NT127copZ3   DAGFDATE* 68
NT127copZ2   DAGFDATE* 68
RdAWcopZ1    DAGFDATE* 68
RdAWcopZ2    DAGFDATE* 68
RdAWcopZ3    DAGFDATE* 68
RdAWcopZ4    DAGFDATE* 68
RdAWcopZ5    DAGFDATE* 68
RdAWcopZ6    DAGFDATE* 68
             ********
```

Fig. S4. CopZ alignment in NT127 and RdAW. Alignment of the three and six copies of CopZ in NT127 and RdAW, respectively. Metal-binding motif CXXC found in metallochaperones is boxed (2-5). Percent identity is at least 97% between CopZ copies in NT127 and RdAW with three amino acid substitutions amongst the two strains that are either conservative or semi- conservative (amino acid differences are shaded). Rd KW20 HI0292 and HI0291 CopZ are identical in amino acid sequence to RdAW copZ1 and copZ3-6, respectively.

REFERENCES

1. Gawronski JD, Wong SM, Giannoukos G, Ward DV, Akerley BJ. 2009. Tracking insertion mutants within libraries by deep sequencing and a genome-wide screen for *Haemophilus* genes required in the lung. Proc Natl Acad Sci U S A 106:16422-7.

2. Banci L, Bertini I, Del Conte R, Markey J, Ruiz-Dueñas FJ. 2001. Copper trafficking: the solution structure of Bacillus subtilis CopZ. Biochemistry 40:15660-8.

3. Radford DS, Kihlken MA, Borrelly GP, Harwood CR, Le Brun NE, Cavet JS. 2003. CopZ from Bacillus subtilis interacts in vivo with a copper exporting CPx-type ATPase CopA. FEMS Microbiol Lett 220:105-12.

4. Zhou L, Singleton C, Le Brun NE. 2008. High Cu(I) and low proton affinities of the CXXC motif of Bacillus subtilis CopZ. Biochem J 413:459-65.

5. Robinson NJ, Winge DR. 2010. Copper metallochaperones. Annu Rev Biochem 79:537-62.

Table S1. Primers used in the study.

Primer	Sequence
CuChapSeq_F	5'AACCCAACATTGCGTGAGTGTC
CuChapSeq_R	5'CTTGCTTGCGTAGCGTCAAATAC
5CuATPase1	5'TGACGAATGAACGCACTCGC
3CuATPase1pG	5'AGGCTTATGTCAATTCGAGAATTGTTAGTTGTTTTATTGATGGTT AAAC
5pGent1	5'CAATTCTCGAATTGACATAAGCCT
3Gent2	5'TTAGGTGGCGGTACTTGGGTCGAT
5CuATPase2pG	5'ATCGACCCAAGTACCGCCACCTAATTTCATTTATTTTTATACGCA ATCGGTG
3CuATPase2	5'ATAATACGGCACTATTCCATTCAGG
CuATP_comp_F	5'AAAGCTCTTCAATGCTTCTTGACCTTAACCTTATGTAAAGGTTTA TAGT
CuATP_comp_R	5'AAAGCTCTTCATTAGAACCGCACTTTTTTCAATCTTAAGGC
3HI0290_1pG	5'AGGCTTATGTCAATTCGAGAATTGTTAGATATGGGCAGAGAAACCTGTTT TATG
x-xylF	5'GAACCGCAATTTCTGCCGCTTTATC
HI0290_comp_R	5'ACTCTGTGGCGTCAAATCCAGCATTTTTACTCCTTGTTAGTTGTTTTATT GATGG
HI0290_comp_F	5'ATGCTGGATTTGACGCCACAGAGTAAAAAAATTTCAATTCAGATTGGTGG GATGA
3gent2_290tail	5'ACTCTGTGGCGTCAAATCCAGCATTTAGGTGGCGGTACTTGGGTCGAT
q3ATPase	5'GGGTGCTTTTGAACCTTGTGC
Chap_comp_R	5'AAAGCTCTTCTTTACTCTGTGGCGTCAAATCCAGCATCTTC
5cueR1	5'CGAATACTACCGATTGCTCCGC
3cueR1pG	5'AGGCTTATGTCAATTCGAGAATTGCATAAAAAACTTCTTGACCTTAACCT TAC
5cueR2pG	5'ACCCAAGTACCGCCACCTAAGTGAAAAGTGCGGTTAATATTCC
3cueR2	5'TCTAAGCCCATTTGCTCCTCG
cueR_comp_F	5'GATGCTCTTCAATGTTACTCCTTGTTAGTTGTTTTATTGATGG
cueR_comp_R	5'AAAGCTCTTCCTTATCCGTTCAAGCCATTCAAAATTAAAC
3xylFcop	5'TGATGGCTACACTATAAACCTTTACGTAAGGTTAAGGTCAAGAAGTTTTT TATGAAAATCAAATCAGCTTTACTTACC
5copLacZ	5'CGTAAAGGTTTATAGTGTAGCCATCAATAAAACAACTAACAAGGAGTAAA AATGACCATGATTACGGATTCACT
lacZtrcKan	5'AAGCATTGGTGCACCGTGCAGTCGTTATTTTTGACACCAGACCAACTGGTA
IFTrcF	5'CGACTGCACGGTGCACCAATGCTT
x-xylB2	5'GCCATAGCGAAGCGTAAAAGTAGCAC
XT10thyA-F	5'AGGGCTTGAATCGCACCTCCA
pXGPseqF	5'CTTTGGAATGGGCAGAACTAC
lac7	5'TCGATAATTTCACCGCCGAAAGG
q5MerR	5'GAGCAGTTAGCACTTTCACTTCTCG
5merR1	5'CGAATACTACCGATTGCTCCGC
